# Long-term visual outcomes in pachychoroid spectrum diseases and its associating factors of eyes with chronic central serous chorioretinopathy

**DOI:** 10.1038/s41598-023-49153-7

**Published:** 2023-12-11

**Authors:** Keiko Azuma, Nobuya Tanaka, Shuichiro Aoki, Kohdai Kitamoto, Kohei Ueda, Tatsuya Inoue, Ryo Obata

**Affiliations:** 1 Department of Ophthalmology, Graduate School of Medicine and Faculty of Medicine, The University of Tokyo, Tokyo, 113-8655 Japan; 2 Department of Ophthalmology and Micro-Technology, Yokohama City University School of Medicine, 4-57 Urafune-cho, Minami-ku, Yokohama, Kanagawa 232-0024 Japan

**Keywords:** Medical research, Signs and symptoms

## Abstract

To analyze the long-term visual outcomes of pachychoroid spectrum diseases (PSD). Retrospective study. We reviewed the medical charts of consecutive patients with PSD, including focal choroidal excavation (FCE), pachychoroid pigment epitheliopathy (PPE), central serous chorioretinopathy (CSC), and pachychoroid neovasculopathy (PNV). The patients initially visited the Tokyo University Hospital from January 2008 to March 2021. Survival analyses were performed, in which loss of vision was defined as visual acuity (VA) of 0.2 logarithm of minimal angle of resolution (logMAR) or worse, 0.5 logMAR or worse, or VA worsening by 0.3 logMAR or greater. Moreover, we further investigated factors associated with visual prognosis, particularly in the CSC group. A total of 741 eyes of 638 patients were included in this analysis. The CSC or PNV group showed significantly worse visual prognosis than the FCE&PPE group for VA to 0.2 logMAR or worse (P = 0.0117 or 0.0001, respectively) and for VA worsening by 0.3 logMAR or greater (P = 0.0283 or 0.0037, respectively). In the CSC group, unlike age, sex, or treatment history, the accumulative duration of subfoveal fluid existence ≥ 12 months (continuous or intermittent) was significantly associated with visual prognosis (P < 0.0001). Among PSD, CSC and PNV were associated with a higher risk of vision loss in the long term than FCE and PPE. The duration of subretinal fluid existence was identified as a significant factor affecting long-term visual outcomes in CSC.

## Introduction

Pachychoroid spectrum diseases (PSD) were first reported in 2015. Numerous studies have been performed on these diseases, particularly in Asian countries^1–3^. The classification of these diseases is undergoing continuous development. In 2019, Cheung et al. suggested that PSD should include pachychoroid pigment epitheliopathy (PPE), focal choroidal excavation (FCE), central serous chorioretinopathy (CSC), and pachychoroid neovasculopathy (PNV). The most commonly shared characteristics among these phenotypes are increased choroidal thickness due to dilated veins (pachyvessels) in the Haller’s layer and choroidal vascular hyperpermeability (CVH) on indocyanine green angiography (ICGA)^2–6^.

FCE has been defined as an area of concavity in the choroid, typically in the macular region and visible through optical coherence tomography (OCT) imaging. CSC and PPE are characterized by pigmentary changes in the macular area with and without exudative changes, respectively, while PNV presents macular choroidal neovascularization^1^. On ICGA, pachyvessels appear as a cluster of relatively straight and dilated choroidal vessels. In addition to choroidal venous dilatation, choroidal filling defects, delayed arterial filling in the early phase, and focal or punctate hyperfluorescence have been observed in eyes with CSC, PNV, and FCE. These findings are suggestive of possible choroidal ischemia^3,7–9^.

It has been reported that some types of PSD (e.g., PPE and FCE) are linked to relatively better preservation of vision than other types^4,10,11^. However, another report indicated that PNV causing exudative change by macular neovascularization (MNV) resulted in a marked loss of vision^1,11,12^.

Regarding CSC, it was reported that long-term visual outcomes are generally favorable even in the chronic type^13,14^. Research has also demonstrated that chronic CSC (cCSC) represents progressive chorioretinopathy, with many patients with cCSC experiencing significant vision loss and lower vision-related quality of life^1,4,15,16^.

Previous studies have investigated the pathophysiology, classification, phenotypes, clinical features, imaging characteristics, and management of PSD. However, thus far, research has not focused on the long-term visual outcomes of each subtype of PSD in real-world clinical practice.

Therefore, the aim of this study was to evaluate the long-term visual outcomes of each subtype of PSD, particularly focusing on CSC, for which controversial prognoses have been reported.

## Methods

This retrospective study was conducted according to the tenets of the Declaration of Helsinki and was approved by the Institutional Review Board of the University of Tokyo (Tokyo, Japan). Owing to the retrospective nature of the study, the Institutional Review Board waived the requirement for written informed consent. Nonetheless, patients who did not authorize the use of their medical records for research purposes were excluded from the analysis.

We retrospectively reviewed the medical charts of consecutive patients who initially visited the University of Tokyo Hospital from January 2008 to March 2021, underwent dye angiography including ICGA, and were diagnosed with age-related macular degeneration, CSC, retinal pigment epithelial atrophy, or FCE in either eye. After multimodal images including OCT and ICGA of both eyes in each patient were evaluated by the retinal specialists (KA, NT, and RO), the eyes with characteristic findings of pachychoroid diseases were included in the study. All patients underwent a standard examination that included measurement of best-corrected visual acuity, slit-lamp biomicroscopy, funduscopy, and spectral domain-OCT (Spectralis; Heidelberg Engineering, Heidelberg, Germany) at each visit. OCT angiography was performed to exclude the development of MNV. Fundus autofluorescence imaging (Heidelberg Retina Angiograph 2; Heidelberg Engineering) was performed to detect atrophic changes in retinal pigment epithelium. Best-corrected visual acuity was measured using the Landolt C chart, and values were converted into logarithm of minimal angle of resolution (logMAR). All patients underwent fluorescein angiography and ICGA at the time of initial treatment, unless contraindicated. The inclusion criterion was diagnosis with CSC with CVH lesions in the examined eye. The eyes without serous detachment at the time of the baseline were excluded from the analysis. Patients complicated with other retinal diseases (e.g., diabetic retinopathy, retinal vascular diseases, myopic maculopathy, glaucoma, and significant cataract that could affect visual function) were excluded. All these examinations were performed within one week after initial presentation.

### Diagnosis

The recognition of specific clinical and multimodal imaging findings present in eyes with pachychoroid disease continues to evolve^17–19^. In the present study, all patients had at least the pachychoroid phenotype (i.e., reduced fundus tessellation on color fundus photographs, pathologically dilated outer choroidal vessels on OCT and ICGA images, and regional CVH on ICGA images)^20^.

### Treatments

Patients without exudative changes due to PPE and FCE did not receive treatment; however they underwent periodical checkup. For patients with CSC, the attending physician conducted an examination, performed laser photocoagulation, and administered reduced-fluence photodynamic therapy (rfPDT) or treatment with anti-vascular endothelial growth factor (anti-VEGF). The protocol of reduced-fluence PDT with verteporfin (Visudyne; Novartis, Basel, Switzerland) was based on the previous report^21^. Briefly, all patients received a 6 mg/m^2^ infusion of verteporfin over 10 min followed by laser delivery at 689 nm 15 min after the start of the infusion. The treatment for each patient with CSC was selected through the following process. For patients with extrafoveal leakage, laser photocoagulation was recommended. For those with juxta- or sub-fovea leakage, both PDT and anti-VEGF therapy were presented as treatment options. After explaining the details of each treatment, the physician finally determined the treatment considering the patient’s preferences. As for anti-VEGF, the drug was administered once, and patients were followed in the pro re nata protocol with monthly follow-up. Meanwhile, PDT was administered again if exudate persisted or recurred longer than 3 months after the treatment. Patients with PNV were treated with anti-VEGF, occasionally combined with photodynamic therapy, similar to the treatment of neovascular age-related macular degeneration.

### Statistical analysis

We conducted Kaplan–Meier survival analysis for visual prognosis in three groups (i.e., FCE&PPE, CSC, and PNV). The log-rank test was used for comparisons between two groups. In the survival analyses, loss of vision was defined as VA deterioration to 0.2 logMAR or worse (approximately equivalent to the minimum necessary for obtaining a driving license), VA deterioration to 0.5 logMAR or worse (approximately equivalent to “low vision” defined by the World Health Organization), and VA change of 0.3 logMAR or greater (equivalent to three lines changes in vision).

For CSC and PNV, we classified patients according to the initial diagnosis. Additionally, to analyze the visual prognosis of CSC without the development of MNV in more detail, we also used a different classification for similar analyses. The patients who developed PNV during the observation period were included in the PNV group rather than the CSC group. In these cases, the initial date of follow-up was adjusted to the date of initial PNV diagnosis. During the follow-up period, some CSC patients showed the development of PNV. In this report, the group of CSC with the development of PNV was described as CSC(+PNV). On the other hand, the group of CSC who did not show the development of PNV through the follow-up period was described as CSC(−PNV).

Moreover, we investigated factors associated with visual prognosis in the CSC(−PNV) group. Firstly, Kaplan–Meier survival analysis was performed for visual prognosis in five groups classified according to the duration of subfoveal fluid existence (0–3, 3–6, 6–12, 12–24, and > 24 months). Of note, in case of intermittent subfoveal fluid existence, this duration was summed. Secondly, the association between the duration of subfoveal fluid existence (≤ 12 months and > 12 months) or treatment history (no treatment, laser photocoagulation, rfPDT, and anti-VEGF) and visual outcomes was analyzed using the log-rank test. If an eye received multiple treatments, it was classified based on the initial treatment. Thirdly, multivariate analysis was performed to confirm independent associations between the duration of subfoveal fluid existence, treatment history, age, or sex and visual outcome using the Cox proportional hazards regression model.

All statistical analyses were performed using the JMP version 16.0 software (SAS Institute, Cary, NC, USA), and P-values < 0.05 denoted statistically significant differences. All data are expressed as the mean ± standard deviation.

### Ethics approval and consent to participate

The study was conducted in accordance with the tenets of the Declaration of Helsinki and with the approval of the ethics committee at the coordinating center of the University of Tokyo. All patients provided written informed consent prior to participation in the study.

## Results

Background factors for all PSD are presented in Table 1. The mean age of patients in the FCE&PPE and CSC groups was in the 50s, while that of patients in the PNV group was in the 60s. There were statistical differences in the ages at baseline between FCE, PPE, CSC (+PNV), and PNV group (P < 0.0001, ANOVA). FCE or PPE was significantly younger than CSC (+PNV) or PNV (all P < 0.05, Dunnet post hoc analysis). Patients were predominantly male. The PPE and PNV groups had the best and worst mean baseline logMAR acuity (− 0.07 ± 0.07 vs. 0.12 ± 0.23, respectively). There were statistical differences in the Baseline logMAR VA between FCE, PPE, CSC (+PNV), and PNV group (P < 0.0001, ANOVA). FCE or PPE or was significantly better than CSC (+PNV) or PNV (all P < 0.05, Dunnet post hoc analysis). In the CSC(+PNV) group, 28/547 (5%) patients were subsequently diagnosed with PNV; the mean duration from the initial diagnosis of CSC to that of PNV was 4.8 ± 2.8 years (range: 1.0–11.5 years). All patients included in this study were treatment-naive.

**Table 1:** Background factors for all PSD: comparison of visual acuity prognosis among pachychoroid-related diseases by disease type and logMAR visual acuity loss.

Factor	FCE	PPE	FCE&PPE	CSC(+PNV)^a^	PNV^a^	CSC(−PNV)^b^	PNV^b^	P-value
Number of eyes, N	40	39	79	547	115	519	143	
Age (years)	49.5 ± 13.3	53.5 ± 14.6	53.9 ± 13.6	55.3 ± 13.3	65.0 ± 10.5	54.9 ± 13.2	64.2 ± 10.8	< 0.0001
Number of treatment-naive eyes, N	40	39	79	547	115	519	143	
Male, N (%)	23 (58)	31 (79)	54 (68)	389 (71)	85 (74)	375 (72)	101 (71)	0.10
Duration of follow-up from initiation to last visit (months)	63.6 ± 57.6	58.5 ± 12.6	54.0 ± 50.4	55.2 ± 46.4	48.0 ± 38.4	52.5 ± 45.6	58.8 ± 45.6	0.29
Baseline logMAR VA	0.02 ± 0.15	− 0.07 ± 0.07	− 0.02 ± 0.12	0.07 ± 0.24	0.12 ± 0.23	0.06 ± 0.22	0.13 ± 0.28	< 0.0001

Values are presented as the mean ± standard deviation, unless otherwise indicated.

CSC, central serous chorioretinopathy; FCE, focal choroidal excavation; logMAR, logarithm of minimal angle of resolution; MNV, macular neovascularization; PNV pachychoroid neovasculopathy; PPE, pachychoroid pigment epitheliopathy; PSD, pachychoroid spectrum diseases; VA, visual acuity.

^a^Classification of CSC or PNV based on the initial diagnosis.

^b^Patients initially diagnosed with CSC who developed MNV during the follow-up were classified into the PNV group, with the initial visit adjusted to the initial date of PNV diagnosis.

**Figure 1:**
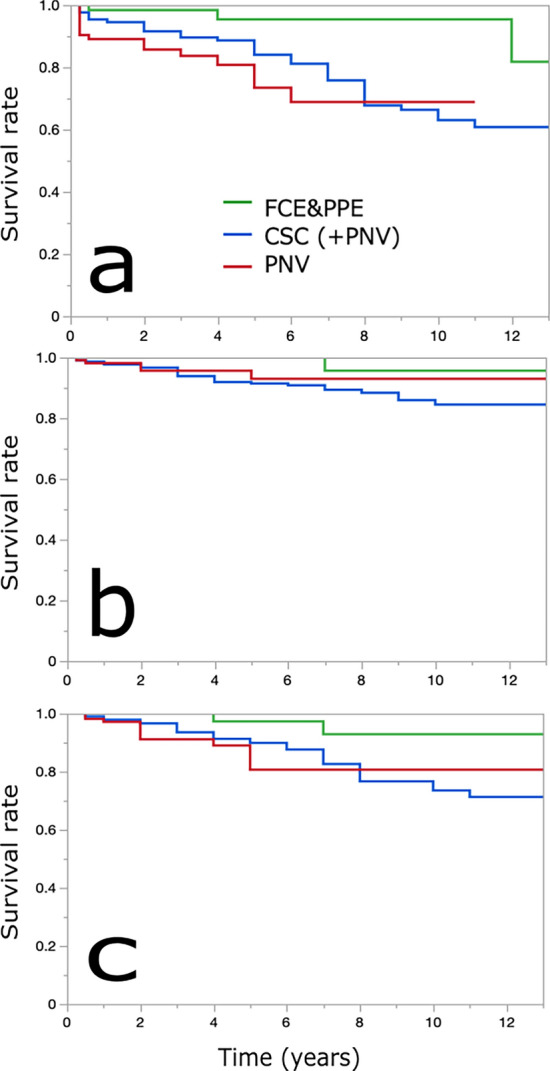
Survival rate estimated using the Kaplan–Meier method for the visual prognosis of all PSD. Green, blue, and red lines indicate the FCE&PPE, CSC(+PNV), and PNV groups, respectively. The CSC(+PNV) and PNV groups exhibited significantly worse survival than the FCE&PPE group for deterioration to 0.2 logMAR or worse (**a**). However, they exhibited similar survival for deterioration to 0.5 logMAR or worse (**b**). Moreover, the CSC(+PNV) or PNV group exhibited worse survival than the FCE&PPE group for a decline of 0.3 logMAR or greater (**c**). CSC, central serous chorioretinopathy; FCE, focal choroidal excavation; logMAR, logarithm of minimal angle of resolution; PNV pachychoroid neovasculopathy; PPE, pachychoroid pigment epitheliopathy; PSD, pachychoroid spectrum diseases.

The visual prognosis was analyzed for all PSD (Table 2, Fig. 1). The CSC(+PNV) or PNV group showed significantly worse survival than the FCE&PPE group for deterioration to 0.2 logMAR or worse. However, the two groups exhibited similar survival for deterioration to 0.5 logMAR or worse. The CSC(+PNV) or PNV group showed worse survival than the FCE&PPE group for 0.3 logMAR decline or greater. Of note, there was no significant difference observed between the CSC(+PNV) and PNV groups in any analysis.

**Table 2:** Survival analysis of visual prognosis for all PSD (FCE&PPE, CSC(+PNV), and PNV).

		FCE&PPE		CSC(+PNV)		PNV	
		Survival rate (%)	No. at risk	Survival rate (%)	No. at risk	Survival rate (%)	No. at risk
logMAR > 0.2	Baseline		79		547		115
	3 years	98	38	90	232	84	42
	5 years	95	28	84	154	73	22
	7 years	95	20	76	91	69	12
P-value*				0.0065		0.0010	
P-value**						NS	
logMAR > 0.5	Baseline		79		547		115
	3 years	100	42	94	309	96	62
	5 years	100	32	91	190	93	36
	7 years	96	23	89	123	93	23
P-value*				NS		NS	
P-value**						NS	
logMAR change > 0.3	Baseline		79		547		115
	3 years	100	42	94	317	91	59
	5 years	97	31	90	195	81	32
	7 years	93	22	83	123	81	19
P-value*				0.024		0.028	
P-value**						NS	

CSC, central serous chorioretinopathy; FCE, focal choroidal excavation; logMAR, logarithm of minimal angle of resolution; NS, not significant; PNV pachychoroid neovasculopathy; PPE, pachychoroid pigment epitheliopathy; PSD, pachychoroid spectrum diseases.

*Compared with FCE&PPE.

**Compared with CSC.

**Figure 2:**
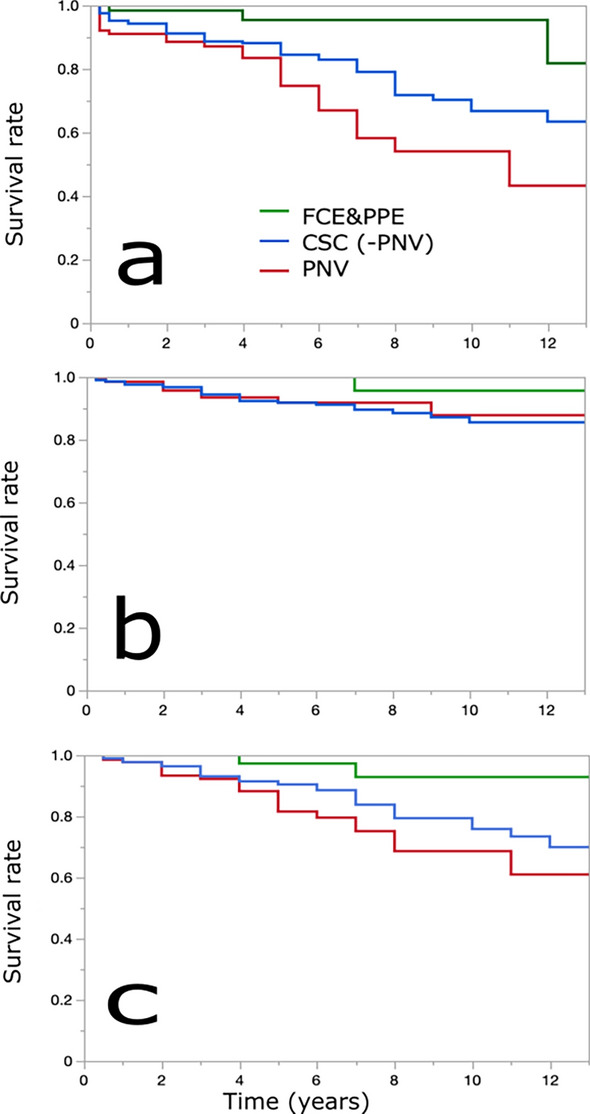
Survival rate estimated using the Kaplan–Meier method for the visual prognosis of all PSD. Green, blue, and red lines indicate the FCE&PPE, CSC(−PNV), and PNV groups, respectively. The CSC(−PNV) and PNV groups exhibited significantly worse survival than the FCE&PPE group for deterioration to 0.2 logMAR or worse (**a**). However, the groups exhibited similar survival for deterioration to 0.5 logMAR or worse (**b**). Moreover, the CSC(−PNV) or PNV group exhibited worse survival than the FCE&PPE group for a decline of 0.3 logMAR or greater (**c**). Significant difference was observed between the CSC(−PNV) and PNV groups for deterioration to 0.2 logMAR or worse. However, the groups exhibited similar results for deterioration to 0.5 logMAR or worse and 0.3 logMAR decline or worse. CSC, central serous chorioretinopathy; FCE, focal choroidal excavation; logMAR, logarithm of minimal angle of resolution; PNV pachychoroid neovasculopathy; PPE, pachychoroid pigment epitheliopathy; PSD, pachychoroid spectrum diseases.

Survival analysis between the FCE&PPE, CSC(−PNV), and PNV groups was performed to analyze the visual prognosis of CSC without the development of MNV in more detail (Table 3, Fig. 2). The CSC(−PNV) or PNV group showed significantly worse survival than the FCE&PPE group for deterioration to 0.2 logMAR or worse. Nevertheless, the groups exhibited similar survival for deterioration to 0.5 logMAR or worse. Moreover, the CSC(−PNV) and PNV groups showed worse survival for 0.3 logMAR decline or greater than the FCE&PPE group. Significant difference was observed between the CSC(−PNV) and PNV groups for deterioration to 0.2 logMAR or worse. Otherwise, these two groups exhibited similar survival.

**Table 3:** Survival analysis of visual prognosis for all PSD (FCE&PPE, CSC(−PNV), and PNV).

		FCE&PPE		CSC(−PNV)		PNV	
		Survival rate (%)	No. at risk	Survival rate (%)	No. at risk	Survival rate (%)	No. at risk
logMAR > 0.2	Baseline		79		519		115
	3 years	98	38	89	217	87	61
	5 years	95	28	84	144	75	38
	7 years	95	20	79	86	58	23
P-value*				0.0012		0.0001	
P-value**						0.016	
logMAR > 0.5	Baseline		79		519		115
	3 years	100	42	94	289	93	86
	5 years	100	32	92	177	92	56
	7 years	96	23	90	113	92	39
P-value*				NS		NS	
P-value**						NS	
logMAR change > 0.3	Baseline		79		519		115
	3 years	100	42	93	293	92	87
	5 years	97	31	90	180	82	53
	7 years	93	22	84	112	75	36
P-value*				0.028		0.0037	
P-value**						NS	

CSC, central serous chorioretinopathy; FCE, focal choroidal excavation; logMAR, logarithm of minimal angle of resolution; NS, not significant; PNV pachychoroid neovasculopathy; PPE, pachychoroid pigment epitheliopathy; PSD, pachychoroid spectrum diseases.

*Compared with FCE&PPE.

**Compared with CSC.

Table 4 shows the mean duration of follow-up, mean baseline VA, duration until treatment, and duration of subretinal fluid existence in patients in each group, categorized by treatment history. There was no acute CSC patient, who showed the resolution of the fluid within three consecutive months^22,23^. In the CSC(−PNV) group, 54% of patients did not receive any treatment. For rfPDT, the longest time to treatment was 25.5 ± 35.0 months. Moreover, the longest total duration of subretinal fluid existence was 8.4 ± 16.0 months. Finally, the longest mean duration of follow-up from initiation to last visit was 81.6 ± 46.8 months. There were statistical differences in the duration of subretinal fluid existence between no treatment, rfPDT, Direct PC, and anti-VEGF group (P = 0.0022, ANOVA). No treatment group was significantly shorter than rfPDT, direct PC, or anti-VEGF (all P < 0.05, Dunnet post hoc analysis).

**Table 4:** Mean duration of follow-up, mean baseline VA, duration until treatment, and duration of subretinal fluid existence in patients in the CSC(−PNV) group categorized by treatment history.

	No treatment	RfPDT	Direct PC	Anti-VEGF	P-value
Number of eyes, N (%)	281 (54)	83 (16)	119 (23)	36 (7)	
Duration of follow-up from initiation to last visit (months)	42.0 ± 43.2	81.6 ± 46.8	66.0 ± 44.4	44.4 ± 30.0	0.06
Baseline logMAR VA	0.05 ± 0.23	0.12 ± 0.26	0.03 ± 0.16	0.12 ± 0.24	
Duration until treatment (months)		25.5 ± 35.0	13.3 ± 25.0	9.8 ± 22.0	< 0.0022
Duration of subretinal fluid existence (months)	2.8 ± 9.1	8.4 ± 16.0	7.6 ± 15.0	6.5 ± 13.4	

Values are presented as the mean ± standard deviation, unless otherwise indicated.

CSC, central serous chorioretinopathy; logMAR, logarithm of minimal angle of resolution; PC, laser photocoagulation; PNV pachychoroid neovasculopathy; RfPDT, reduced-fluence photodynamic therapy; VA, visual acuity; VEGF, vascular endothelial growth factor.

**Figure 3:**
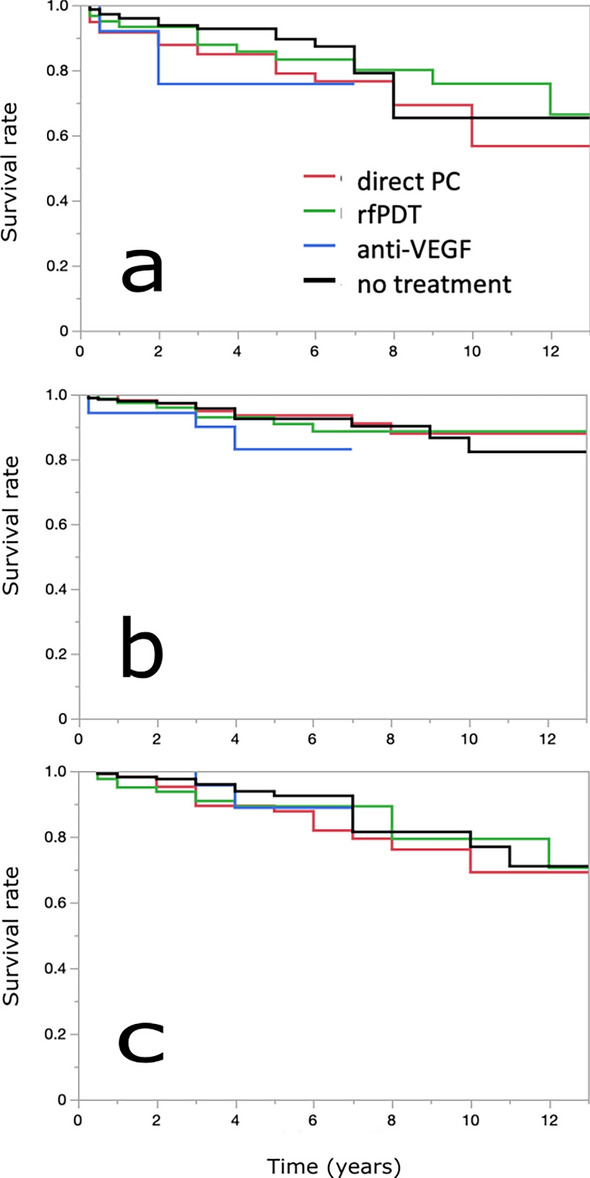
Survival analysis for the comparison of visual acuity prognosis for deterioration to 0.2 logMAR or worse (**a**), 0.5 logMAR or worse (**b**), and a decline of 0.3 logMAR or greater (**c**), based on treatment history in the CSC(−PNV) group. Red, green, blue, and black lines indicate direct laser photocoagulation (PC), reduced-fluence photodynamic therapy (rfPDT), anti-VEGF, and no treatment, respectively. There were no significant differences between the groups of treatment history. CSC, central serous chorioretinopathy; logMAR, logarithm of minimal angle of resolution; PNV pachychoroid neovasculopathy; VEGF, vascular endothelial growth factor.

In the CSC(−PNV) group, comparison of the visual prognosis according to the treatment did not reveal significant differences (Table 5 and Fig. 3).

**Table 5:** Comparison of visual acuity prognosis by treatment history in the CSC(−PNV) group.

		No treatment		RfPDT		Direct PC		Anti-VEGF	
		Survival rate (%)	No. at risk	Survival rate (%)	No. at risk	Survival rate (%)	No. at risk	Survival rate (%)	No. at risk
logMAR > 0.2	Baseline		281		83		119		36
	3 years	93	92	88	51	85	61	76	13
	5 years	90	58	83	35	79	43	76	8
	7 years	79	32	80	26	77	26	76	2
logMAR > 0.5	Baseline		281		83		119		36
	3 years	96	119	93	64	95	84	90	22
	5 years	92	67	91	45	94	56	83	9
	7 years	90	41	89	31	91	38	83	3
logMAR change > 0.3	Baseline		281		83		119		36
	3 years	96	121	91	67	90	82	96	23
	5 years	92	69	89	47	88	54	89	10
	7 years	81	42	89	34	79	33	89	3

CSC, central serous chorioretinopathy; logMAR, logarithm of minimal angle of resolution; PC, laser photocoagulation; PNV pachychoroid neovasculopathy; RfPDT, reduced-fluence photodynamic therapy; VEGF, vascular endothelial growth factor.

**Figure 4:**
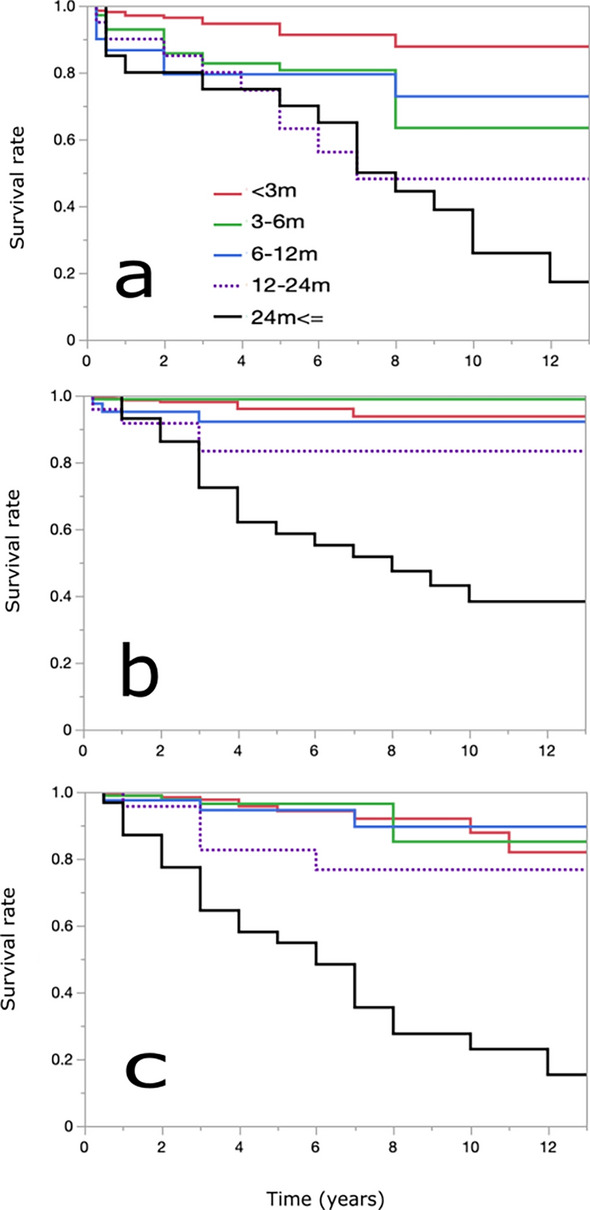
Visual prognosis for deterioration to 0.2 logMAR or worse (**a**), 0.5 logMAR or worse (**b**), and a decline of 0.3 logMAR or greater (**c**) based on the duration of subretinal fluid existence. Red, green and blue lines indicate < 3, 3–6, and 6–12 months, respectively. Purple and black solid lines indicate 12–24 and black line indicate > 24 months. Longer duration was associated with poorer visual outcomes, particularly for those with deterioration lasting ≥ 12 months. logMAR, logarithm of minimal angle of resolution.

Subsequently, we performed Kaplan–Meier survival analysis on visual prognosis for five groups classified by the summed duration of subfoveal fluid existence. The results indicated that longer duration was associated with poorer visual outcome, particularly for those with duration ≥ 12 months (Fig. 1). In the log-rank analysis, patients with duration < 12 months were linked to significantly better visual outcomes than those with duration ≥ 12 months for deterioration to 0.2 logMAR or worse, 0.5 logMAR or worse, and 0.3 logMAR decline or greater (Table 6 and Fig. 4).

**Table 6:** Comparison of visual prognosis by duration of subretinal fluid existence.

		Duration of subretinal fluid existence			
		< 12 months		≥ 12 months	
		Survival rate (%)	No. at risk	Survival rate (%)	No. at risk
logMAR > 0.2	Baseline		463		56
	3 years	90	184	78	33
	5 years	88	116	67	28
	7 years	88	66	49	20
P-value*				< 0.0001	
logMAR > 0.5	Baseline		463		56
	3 years	98	242	77	47
	5 years	97	143	69	34
	7 years	95	86	65	27
P-value*				< 0.0001	
logMAR change > 0.3	Baseline		463		56
	3 years	97	247	72	46
	5 years	95	146	66	34
	7 years	93	87	50	25
P-value*				< 0.0001	

logMAR, logarithm of minimal angle of resolution.

*Compared with < 12 months.

Multivariate analysis confirmed that the duration of subfoveal fluid existence was the only factor significantly associated with visual prognosis in CSC(−PNV) (Table 7).

**Table 7:** Multivariate analysis using the Cox proportional hazards regression model for CSC(−PNV).

Variable	logMAR > 0.2	logMAR > 0.5	logMAR change > 0.3
Age	NS	NS	NS
Sex	NS	NS	NS
Duration of subretinal fluid existence	Estimate: 1.28 95% CI: 0.73–1.81 P < 0.0001	Estimate: 2.23 95% CI: 1.51–3.00 P < 0.0001	Estimate: 2.02 95% CI: 1.44–2.63 P < 0.0001
Treatment history	NS	NS	NS

CI, confidence interval; CSC, central serous chorioretinopathy; logMAR, logarithm of minimal angle of resolution; NS, not significant; PNV, pachychoroid neovasculopathy.

## Discussion

In this study, the long-term visual prognosis of PSD was examined. The FCE&PPE group maintained VA with a 7-year survival rate of approximately 90%, whereas both CSC and PNV groups showed worse visual prognosis. Worse visual prognosis was also shown for patients with CSC without MNV. There was no association observed between treatments and visual prognosis. The duration of subretinal fluid existence was a significant factor affecting visual prognosis in CSC without the development of MNV.

PPE and FCE were associated with the most favorable long-term visual prognosis. In the current analysis, the minimum 7-year visual survival rate was approximately 90%. Using a large cohort of patients, Yagi et al.^20^ revealed a relatively favorable natural course of PPE. The long-term analysis showed that 16.8% of PPE eyes developed CSC during the 6-year follow-up period^20^. A Turkish study with a mean follow-up of 5.2 years reported that 17.6% of patients with PPE (46 eyes of 44 patients) developed CSC. Nevertheless, there was no development of PNV in any of the studied eyes^24^. Regarding FCE, despite the lack of robust epidemiological data, FCE is generally associated with favorable long-term visual outcomes, unless the patients develop MNV^11,25^. According to the results of the present and previous studies, PPE and FCE appear benign disorders that typically do not affect VA, despite their occasional progression to CSC or PNV^6,26^.

Regarding the visual course of PNV, the presence of MNV exudates has been associated with severe reductions in vision^1,11,12^. The results of the present study showed that a quarter of patients with PNV experienced visual decline of 0.3 logMAR or greater in 7 years. This findings support the worse visual prognosis linked to PNV versus PPE or FCE.

Nevertheless, the visual prognosis of CSC remains controversial. It has been reported that the long-term visual outcomes of cCSC are generally favorable, and approximately 55% of patients maintain better than 20/40 vision in at least one eye after 10 years of disease. Of note, 79.7% of patients in the cohort met the visual standard to qualify for a driver’s license at the final visit, and only a small proportion of patients (12.8%) were deemed legally blind at the final visit^13^. Moreover, Breukink et al., indicated that cCSC is a progressive disease in many patients. This causes a decline in VA over time, which is accompanied by lower vision-related quality of life^15^. The results of the present study suggest that the prognosis of CSC is almost comparable to that of PNV. This may be attributed to three reasons. Firstly, the development of PNV causes similar visual decline to that noted in patients with PNV at the first visit. Secondly, CSC is associated with poor visual prognosis due to exudation, even in the absence of MNV. Finally, CSC is linked to poor visual prognosis, particularly in patients treated with specific therapy for exudation. Next, we performed survival analysis between the FCE&PPE, CSC(–PNV), and PNV groups to determine the visual prognosis of CSC without the development of MNV. The results showed that the prognosis of CSC was worse than that of FCE&PPE, even in patients without MNV. Additionally, there was no association observed between treatment and visual prognosis in CSC. Studies have revealed that VA reduction is independent of treatment^13,27^. Our findings are consistent with those previously reported, suggesting that exudative changes induced by CSC can lead to visual decline regardless of the administered treatment.

In this study, we also focused on the duration of subfoveal fluid existence as a potential indicator of poor visual prognosis. The survival and multivariate Cox proportional hazards regression analyses revealed that the duration of subfoveal fluid existence was a significant factor affecting visual prognosis in CSC without the development of MNV. A previous study of 43 patients with an average follow-up of 22.8 months showed that shorter periods of subfoveal fluid existence were correlated with better VA than that of longer periods^28^. However, the shortest duration which has a clinically significant impact on VA remains unclear^29^. The present study, which included 519 eyes and involved an average follow-up period of 53 months, supported the findings of previous investigations with larger sample sizes and longer follow-up periods. Additionally, the present data suggested that the duration of 1 year is an important time limit. Of note, for patients with intermittent fluid existence, the duration of subfoveal fluid existence was summed. In clinical practice, for CSC, 3 or 6 months of fluid existence allows changes in the strategy for observation and intervention. If the fluid disappears during this period, observation is typically continued. In such cases, the association of intermittent fluid with visual prognosis remains unclear. The results of the present study indicated that subfoveal fluid existence (persistent or intermittent) may influence long-term visual prognosis when the integrated duration exceeds 1 year.

Strengths of the present study include its relatively large sample size, detailed multimodal imaging, and long-term follow-up. However, there are certain limitations in this investigation. Firstly, this study was retrospective and biases cannot be excluded. Therefore, a prospective study is needed, though it may be difficult to obtain such a large sample in real-world clinical practice. Secondly, we utilized the date when PSD were identified through multimodal imaging as the date of their incidence. However, we did not determine the duration of disease before the patients visited our clinic. Considering that our clinic is a tertiary referral center, the actual duration might be longer than that reported in the results. Third, during the period when the patients selected, the concept of PSD may not have been established. However, we have revised the definition criteria for PSD according to the most recent research.

In conclusion, this study evaluated the long-term visual outcomes of different PSD. CSC and PNV were associated with worse visual prognosis versus FCE&PPE. The duration of subretinal fluid existence, rather than treatment, was identified as a significant factor associated with visual prognosis in CSC. Although the current results cannot be generalized to all eyes with PSD, this study assessed the visual prognosis of PSD in Asian patients. Hence, the present findings may have important clinical implications.

## Data Availability

The datasets generated and/or analyzed during this study will be made available by the corresponding author upon reasonable request.
